# Is wet swab superior to dry swab as an intranasal screening test?

**DOI:** 10.1186/2052-0492-1-10

**Published:** 2013-11-27

**Authors:** Hideharu Hagiya, Mitsunobu Mio, Tomoko Murase, Keiko Egawa, Yumi Kokumai, Taeko Uchida, Naoki Morimoto, Fumio Otsuka, Sumiko Shiota

**Affiliations:** Department of General Medicine, Okayama University Graduate School of Medicine, Dentistry and Pharmaceutical Sciences, 2-5-1 Shikata-cho, Kitaku, Okayama, 700-8558 Japan; Emergency Unit and Critical Care Center, Tsuyama Central Hospital, Okayama, 708-0841 Japan; Laboratory of Pharmacology, School of Pharmacy, Shujitsu University, Okayama, 703-8516 Japan; Department of Clinical Laboratory, Microbiology Division, Tsuyama Central Hospital, Okayama, 708-0841 Japan; Department of Nursing, Tsuyama Central Hospital, Okayama, 708-0841 Japan; Laboratory of Pathogenic Microbiology, School of Pharmacy, Shujitsu University, Okayama, 703-8516 Japan

**Keywords:** Intensive care unit, Methicillin-resistant *Staphylococcus aureus*, Screening test, Swab

## Abstract

Methicillin-resistant *Staphylococcus aureus* is still a great concern, and recognition of the carrier is essential for appropriate infection control in intensive care units. The utility of wet swab compared to dry swab as an intranasal screening test has not been well assessed yet. A comparative study of the wet and dry swab in its ability to detect the organism was performed against critically ill patients, and it was found that there were no statistically significant differences between the two different methods. The wet swab did not show increased sensitivity compared to dry one.

## Correspondence/findings

Nosocomial infection by methicillin-resistant *Staphylococcus aureus* (MRSA) is a great concern in the intensive care unit (ICU) where critically ill patients are gathered. However, controlling MRSA infection is still a hard matter [1]. Since bacterial culture of nasal sample is inexpensive, easy, and available, it is a standard method for the screening test for MRSA carrier. An earlier study comparing the sensitivities of dry and wet swab sampling has shown that these methods led to the same results regarding the detection of intranasal MRSA [2]. However, it is possible that the effectiveness of swab-screening tests may be differed depending on the sampling technique, condition, and the circumstances of each ICU. We therefore conducted a prospective study in purpose of reassessing the validity of wet swab as an intranasal screening test for MRSA carrier in our clinical setting.

The study was performed at an ICU of Tsuyama Central Hospital (Okayama, Japan) from March to May in 2012. Only those patients who were provided with informed consent were eligible for inclusion. For each patient, ICU nurse obtained two anterior nares samples (one dry and one wet), using rayon swab (CultureSwab Plus: Becton, Dickinson and Company, BBL). A naris for sampling was chosen in a random manner for wet swab and the other for dry. Wet swabs were manually moistened with sterile saline just before sampling. After sampling, the specimen was immediately transferred to the own microbiology division. The samples were plated on mannitol salt agar plate after washing the swabs with 1 mL of sterile saline. After incubating 24 h at room temperature, the number of colonies was counted, and the identification of the organism was performed using Microscan WalkAway® (Siemens, Tokyo, Japan). The comparison between the number of colonies of MRSA, methicillin-sensitive *S. aureus* (MSSA), and all bacteria grown on the plate was performed. Statistical analysis was performed using Kaleida Graph 4.1 (Synergy Software, Reading, PA, USA), and Wilcoxon signed rank test was applied. The present study protocol (No. 122) was approved by ethics committee of Tsuyama Central Hospital.

**Figure 1:**
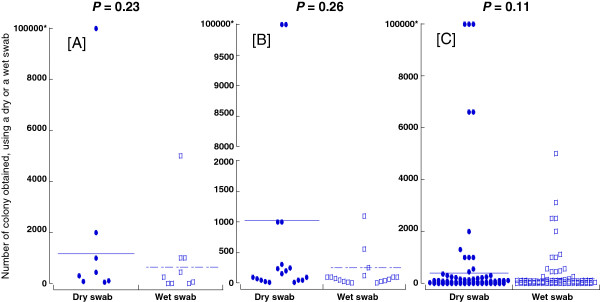
**Comparison between the numbers of colonies obtained from nasal cavities, using a dry swab and a wet swab. A** Methicillin-resistant *Staphylococcus aureus* (MRSA) (*n* = 12), **B** methicillin-sensitive *S. aureus* (*n* = 23), **C** all bacteria (*n* = 141). The horizontal line expresses the average. Asterisk ‘*’ indicates the countless number of colonies was considered to be 10,000.

The total number of subjects was 141. MRSA was isolated from eight samples with dry swab and nine samples with wet swab, and MSSA was isolated from 18 samples with dry swab and 17 samples with wet swab, respectively. Comparison of dry and wet swab was performed in those MRSA positive (A), MSSA positive (B), and all bacteria (C) (Figure 1). There were no statistically significant differences between dry swab and wet swab in each group ((A) *P* = 0.23, (B) *P* = 0.26, and (C) *P* = 0.11).

MRSA can be easily transferred by healthcare workers [3] and often causes life-threatening infections in ICU [4]. Isolating or cohort of those carrier patients is generally recommended [5], and a recent study reported that the rate of MRSA infections was reduced by 62% in ICU and 45% in the other ward with an introduction of MRSA prevention bundle [6]. On the other hand, Huskins et al. reported that surveillance for MRSA colonization combined with subsequently expanded barrier precautions was not effective in reducing the transmission of MRSA [7]. The recognition of MRSA carrier in ICU is, however, considered essential for the infection control, and for that, a high-quality screening method is indispensable [8, 9].

The efficacy of progressive screening test on nasal swab for MRSA with using polymerase chain reaction (PCR) has been reported [10]. PCR screening outperforms the classical bacterial culture in its high sensitivity and specificity, but it cannot be introduced to majority of medical institutions because of its high cost and equipment investments.

Recent studies showed that universal decolonization using chlorhexidine was more effective than ‘screening and isolation’ strategy in reducing MRSA infection rate [11, 12]. However, such a methodology is still not appreciated as a general way to control nosocomial MRSA infection, and we consider that establishment of a reliable screening method is fundamental at present.

Provided that the wet swab yielded more sensitivity in detecting the pathogen, it would be appreciated since it is easy and available method, and does not cost. However, according to our result, the wet swab did not show increased sensitivity compared to the dry one. This result was same as the previous study [2]. The sampling protocol in our study, inserting the wet and dry swabs into each nostril separately, could have influenced the result. According to Kildow et al., healthy adults are more likely to carry *S. aureus* in one nostril than in both [13]. Or, since the distinctive sampling method was not defined precisely in our protocol, therefore the depth or degree of swab insertion into nasal cavities could be different in each subject, which could lead to the sampling error. Small sample size could also be responsible to the result. In any cases, our result indicates that the validity of preparing wet swab rather than dry one is not warranted as an intranasal screening test for MRSA carrier in ICU.

## Abbreviations

ICU: Intensive care unit
MRSA: Methicillin-resistant *Staphylococcus aureus*
MSSA: Methicillin-sensitive *S. aureus*
PCR: Polymerase chain reaction.

## References

[1] Muto CA, Jernigan JA, Ostrowsky BE, Richet HM, Jarvis WR, Boyce JM, Farr BM (2003). SHEA: SHEA: SHEA guideline for preventing nosocomial transmission of multidrug-resistant strains of Staphylococcus aureus and enterococcus. Infect Control Hosp Epidemiol.

[2] Codrington L, Kuncio D, Han J, Nachamkin I, Tolomeo P, Hu B, Lautenbach E (2013). Yield of methicillin-resistant Staphylococcus aureus on moist swabs versus dry swabs. Am J Infect Control.

[3] Morgan DJ, Rogawski E, Thom KA, Johnson JK, Perencevich EN, Shardell M, Leekha S, Harris AD (2012). Transfer of multidrug-resistant bacteria to healthcare workers' gloves and gowns after patient contact increases with environmental contamination. Crit Care Med.

[4] Boucher H, Miller LG, Razonable RR (2010). Serious infections caused by methicillin-resistant Staphylococcus aureus. Clin Infect Dis.

[5] Lee BY, Singh A, Bartsch SM, Wong KF, Kim DS, Avery TR, Brown ST, Murphy CR, Yilmaz SL, Huang SS (2013). The potential regional impact of contact precaution use in nursing homes to control methicillin-resistant Staphylococcus aureus. Infect Control Hosp Epidemiol.

[6] Jain R, Kralovic SM, Evans ME, Ambrose M, Simbartl LA, Obrosky DS, Render ML, Freyberg RW, Jernigan JA, Muder RR, Miller LJ, Roselle GA (2011). Veterans Affairs initiative to prevent methicillin-resistant Staphylococcus aureus infections. N Engl J Med.

[7] Huskins WC, Huckabee CM, O’Grady NP, Murray P, Kopetskie H, Zimmer L, Walker ME, Sinkowitz-Cochran RL, Jernigan JA, Samore M, Wallace D, Goldmann DA (2011). STAR*ICU Trial Investigators: STAR*ICU trial investigators: intervention to reduce transmission of resistant bacteria in intensive care. N Engl J Med.

[8] Kuehnert MJ, Kruszon-Moran D, Hill HA, McQuillan G, McAllister SK, Fosheim G, McDougal LK, Chaitram J, Jensen B, Fridkin SK, Killgore G, Tenover FC (2006). Prevalence of Staphylococcus aureus nasal colonization in the United States, 2001–2002. J Infect Dis.

[9] Graham PL, Lin SX, Larson EL (2006). A US population-based survey of Staphylococcus aureus colonization. Ann Intern Med.

[10] Robotham JV, Graves N, Cookson BD, Barnett AG, Wilson JA, Edgeworth JD, Batra R, Cuthbertson BH, Cooper BS (2011). Screening, isolation, and decolonisation strategies in the control of methicillin resistant Staphylococcus aureus in intensive care units: cost effectiveness evaluation. BMJ.

[11] Huang SS, Septimus E, Kleinman K, Moody J, Hickok J, Avery TR, Lankiewicz J, Gombosev A, Terpstra L, Hartford F, Hayden MK, Jernigan JA, Weinstein RA, Fraser VJ, Haffenreffer K, Cui E, Kaganov RE, Lolans K, Perlin JB, Platt R, The CDC (2013). Prevention Epicenters Program, The AHRQ DECIDE Network and Healthcare-Associated Infections Program: targeted versus universal decolonization to prevent ICU infection. N Engl J Med.

[12] Kildow BJ, Conradie JP, Robson RL (2002). Nostrils of healthy volunteers are independent with regard to Staphylococcus aureus carriage. J Clin Microbiol.

[13] Climo MW, Yokoe DS, Warren DK, Perl TM, Bolon M, Herwaldt LA, Weinstein RA, Sepkowitz KA, Jernigan JA, Sanogo K, Wong ES (2013). Effect of daily chlorhexidine bathing on hospital-acquired infection. N Engl J Med.

